# Demographic and regional trends in Nonmelanoma skin cancer mortality in the United States, 1999–2020

**DOI:** 10.1186/s12885-026-15953-z

**Published:** 2026-03-31

**Authors:** Ahsan Raza Raja, Zoha Zahid Fazal, Aisha Sethi

**Affiliations:** 1 Department of Medicine, Aga Khan University, Stadium Road, P. O. Box 3500, Karachi, Sindh 74800 Pakistan; 2 Stanford University School of Medicine, Palo Alto, CA USA; 3 Department of Dermatology, Yale University School of Medicine, New Haven, CT USA; 4 Yale Dermatology Global Health Program, New Haven, CT USA

**Keywords:** Demographic trends, Regional variation, Nonmelanoma skin cancer, Mortality, United States, Age-adjusted mortality, Joinpoint regression, Epidemiology

## Abstract

**Background:**

Nonmelanoma skin cancer (NMSC) is the most common malignancy worldwide, yet the United States lacks a dedicated registry to monitor its mortality. We aimed to characterize temporal trends and demographic and regional disparities in age-adjusted NMSC mortality in the U.S. from 1999 to 2020.

**Methods:**

We conducted a retrospective analysis of CDC WONDER (Centers for Disease Control and Prevention Wide-Ranging Online Data for Epidemiologic Research) database from 1999 to 2020 for NMSC. Age-adjusted mortality rates (AAMRs) per 100,000 persons and annual percent change (APC) were calculated and stratified by year, sex, race, age group, and geographic region.

**Results:**

A total of 68,223 NMSC-related deaths occurred. Overall AAMR increased from 0.845 (95% CI 0.810–0.879) in 1999 to 1.002 (0.971–1.032) in 2020. Trends showed an initial decline (1999–2004 APC − 0.7%), followed by a rise (2004–2020 APC 1.6%). Men had higher mortality than women (1.55 vs. 0.43 per 100,000), with significant increases in both sexes (men 1999–2016 APC 1.4%; women 2011–2020 APC 2.5%). NH White individuals exhibited the highest AAMR (1.03) and steepest rise (2005–2015 APC 2.8%), while NH Blacks declined (APC − 1.5%) and Hispanics increased modestly (APC 0.9%). Those ≥ 65 years had the highest AAMR (5.63). Regionally, the South (1.01) and rural areas (1.04) bore the greatest burden, with persistent increases (rural APC 1.7%; suburban APC 1.4%).

**Conclusions:**

Despite diagnostic and therapeutic advances, U.S. NMSC mortality has risen, with marked demographic and geographic disparities. Enhanced mortality surveillance, targeted prevention efforts, and equitable access to dermatologic care are needed to mitigate this growing public health burden.

**Supplementary Information:**

The online version contains supplementary material available at 10.1186/s12885-026-15953-z.

## Background

Nonmelanoma skin cancer (NMSC) poses a significant burden on global health and the economy, with its incidence rising in aging populations [1]. Representing nearly one-third of all cancers diagnosed annually, NMSC is the most prevalent malignancy worldwide [2]. Within NMSC, basal cell carcinoma (BCC) and squamous cell carcinoma (SCC) account for 99% of tumors and over 5,400 global deaths each month [3]. Ferlay et al. report that estimated NMSC mortality, in absolute numbers, exceeds that of several other malignancies globally, including melanoma [4]. Given NMSC’s generally lower case-fatality relative to melanoma, this likely reflects its extraordinarily high incidence rather than greater lethality. Annual mortality due to NMSC is predicted to increase by at least 50% by 2044 [5]. For example, in Germany, SCC mortality increased fivefold between 2007 and 2016, underscoring how surveillance can reveal rapidly changing mortality patterns and inform targeted prevention and early detection [6].

Over the past decade, novel systemic therapies and artificial intelligence–enabled approaches have transformed NMSC management [7, 8], yet global mortality continues to rise [5]. This paradox may reflect population aging and persistent delays in detection and treatment in subsets of patients. Australia’s comprehensive skin-cancer registry offers a model for surveillance-driven intervention; [9] by contrast, the United States lacks a dedicated NMSC registry and relies on mortality surveillance systems of limited granularity, impeding insight into demographic and regional disparities [10]. To address this gap, we used the Centers for Disease Control and Prevention’s Wide-ranging Online Data for Epidemiologic Research (CDC WONDER) database to evaluate U.S. age-adjusted NMSC mortality trends from 1999 to 2020 using joinpoint modeling, stratified by sex, race/ethnicity, age group, urban–rural classification, and U.S. Census region.

## Methods

### Database, study setting, and population

We extracted U.S. mortality data from the CDC WONDER database for 1999–2020 [11], identifying NMSC-related deaths using ICD‑10 code C44. This publicly available database includes cause-of-death information from death certificates for all 50 states and the District of Columbia and undergoes internal validation to limit misclassification. The CDC WONDER database has been widely used in studies assessing cancers and other causes of mortality. We selected records where NMSC was the underlying cause of death. Institutional review board approval was not required, as CDC WONDER is deidentified and publicly available, and the study adheres to Strengthening the Reporting of Observational Studies in Epidemiology (STROBE) guidelines.

### Data extraction

We extracted data on year of death, population size; place of residence (state and region), place of death (medical facilities [inpatient, outpatient, emergency room, death on arrival, status unknown], home, hospice facility, nursing home/ long-term care, other), urban-rural classification, age at death (< 25, 25–44, 45–64, and 65 + years), sex (male or female), and race/ethnicity (non-Hispanic [NH] White, NH Black, NH Asian, and Hispanic or Latino). To assess the population, the National Center for Health Statistics (NCHS) Urban-Rural Classification Scheme was used which classifies the population into urban (large metropolitan area [population ≥ 1 million]), suburban (medium/small metropolitan area [population 50,000–999,999]), and rural (nonmetropolitan [population < 50,000]) counties per the 2013 U.S. census classification [12]. Regions were defined per U.S. Census Bureau categories: Northeast, Midwest, South, and West [13]. To preserve privacy when death counts are low, the database suppresses data for ages < 25 years and most data for NH American Indians. AAMRs for NH Asians were unreliable in 1999, 2002–2004, and 2008; we estimated those values via linear interpolation and extrapolation.

### Statistical analysis

We calculated age-adjusted mortality rates (AAMRs) per 100,000 population annually and by subgroup, standardizing to the U.S. 2000 population [12]. Crude mortality rates for each year were derived by dividing the total number of NMSC-related deaths by the corresponding U.S. population size. To determine trends in the annual percent change (APC) of AAMRs, we utilized the Joinpoint Regression Program (Version 5.0.2, National Cancer Institute) [14]. The 95% Confidence Intervals (CI) for AAMR were determined at the identified line segments that link join points, using the Monte Carlo permutation tests. This approach allowed us to identify significant changes in AAMR over the period by fitting log-linear regression models wherever temporal variation took place. APCs were treated as increasing or decreasing depending on whether the slope’s change in mortality was significantly different than zero using 2-tailed t-testing. A p-value < 0.05 was deemed statistically significant. All subgroup analyses were performed as separate, single-factor stratifications (e.g., by race/ethnicity or by age group); joint stratification across multiple factors (e.g., age-by-race-by-region) was not performed using these aggregate CDC WONDER outputs.

## Results

A total of 68,223 NMSC-related deaths occurred in the U.S. between 1999 and 2020 (Additional file 1: Supplemental Table 1). Information on the location of death was available for 66,119 (96.9%) of these deaths. Of these, 40.5% occurred at home, 22.9% within medical facilities, 21.4% in nursing homes/long-term care facilities, and 9.4% occurred in hospice facilities (Additional file 1: Supplemental Table 2).

### Annual trends for NMSC-related AAMR

**Fig. 1:**
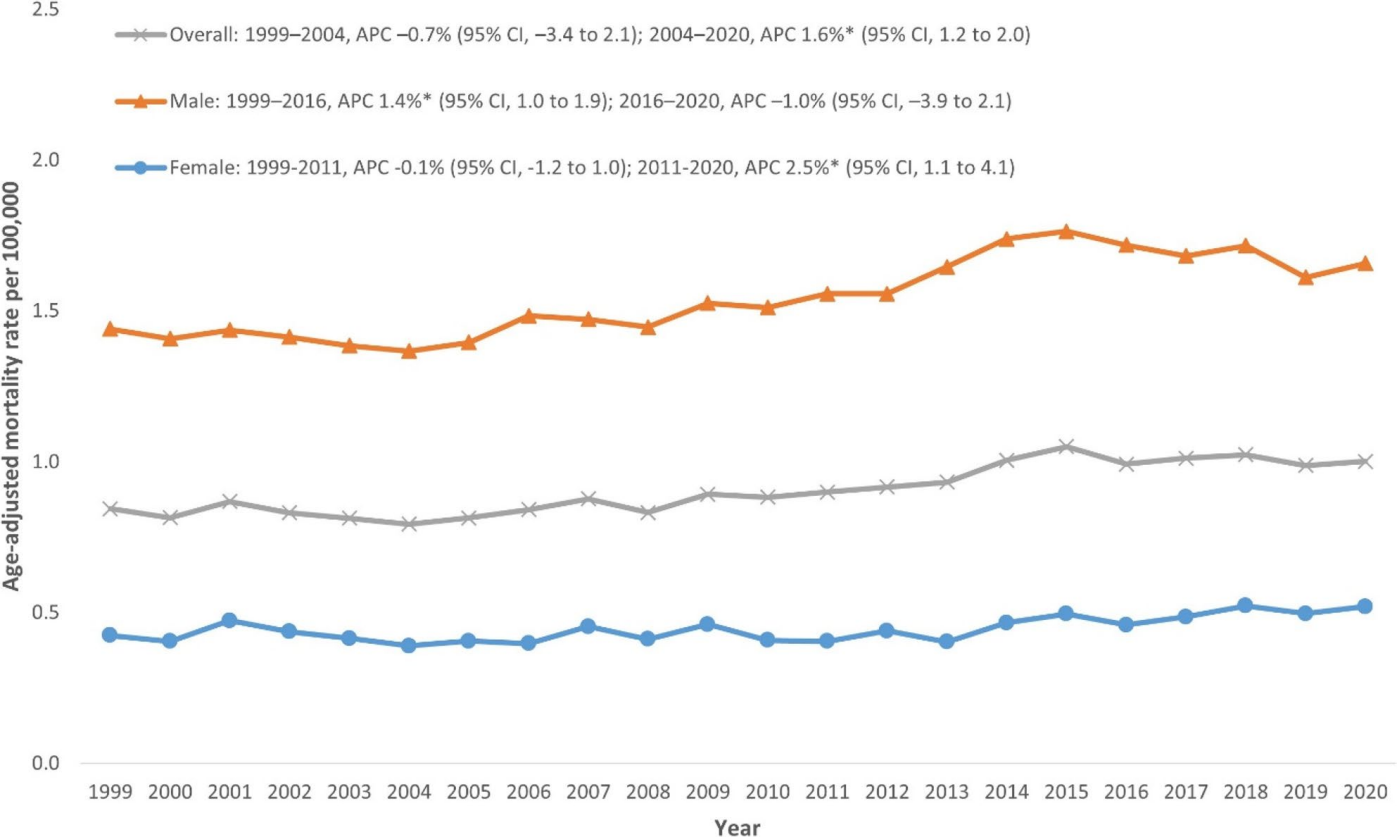
Trends in Nonmelanoma skin cancer-related age-adjusted mortality rate, overall and stratified by sex in the United States, 1999–2020. *Indicates that the annual percentage change (APC) is significantly different from zero at α = 0.05

The AAMR for NMSC-related deaths was 0.845 per 100,000 (95% CI: 0.810–0.879) in 1999 and increased to 1.002 per 100,000 (95% CI: 0.971–1.032) in 2020. Overall, the AAMR showed an initial decline from 1999 to 2004 (APC: -0.7%; 95% CI: − 3.4 to 2.1), followed by a significant rise from 2004 to 2020 (APC: 1.6%; 95% CI: 1.2 to 2.0) (Fig. 1, Additional file 1: Supplemental Table 3).

### NMSC-related AAMR stratified by sex

Men consistently had higher AAMRs than women throughout the study period. The overall AAMR for men was significantly higher at 1.55 per 100,000 compared with women at 0.43 per 100,000. In men, a significant increase from 1999 to 2016 (APC: 1.4%; 95% CI: 1.0 to 1.9) reversed into a declining trend from 2016 to 2020 (APC: -1.0%; 95% CI: − 3.9 to 2.1). Conversely, women exhibited relative stability from 1999 to 2011 (APC: -0.1%; 95% CI: -1.2 to 1.0), but demonstrated a significant increase from 2011 to 2020 (APC: 2.5%; 95% CI: 1.1 to 4.1) (Fig. 1, Additional file 1: Supplemental Table 3).

### NMSC-related AAMR stratified by race

**Fig. 2:**
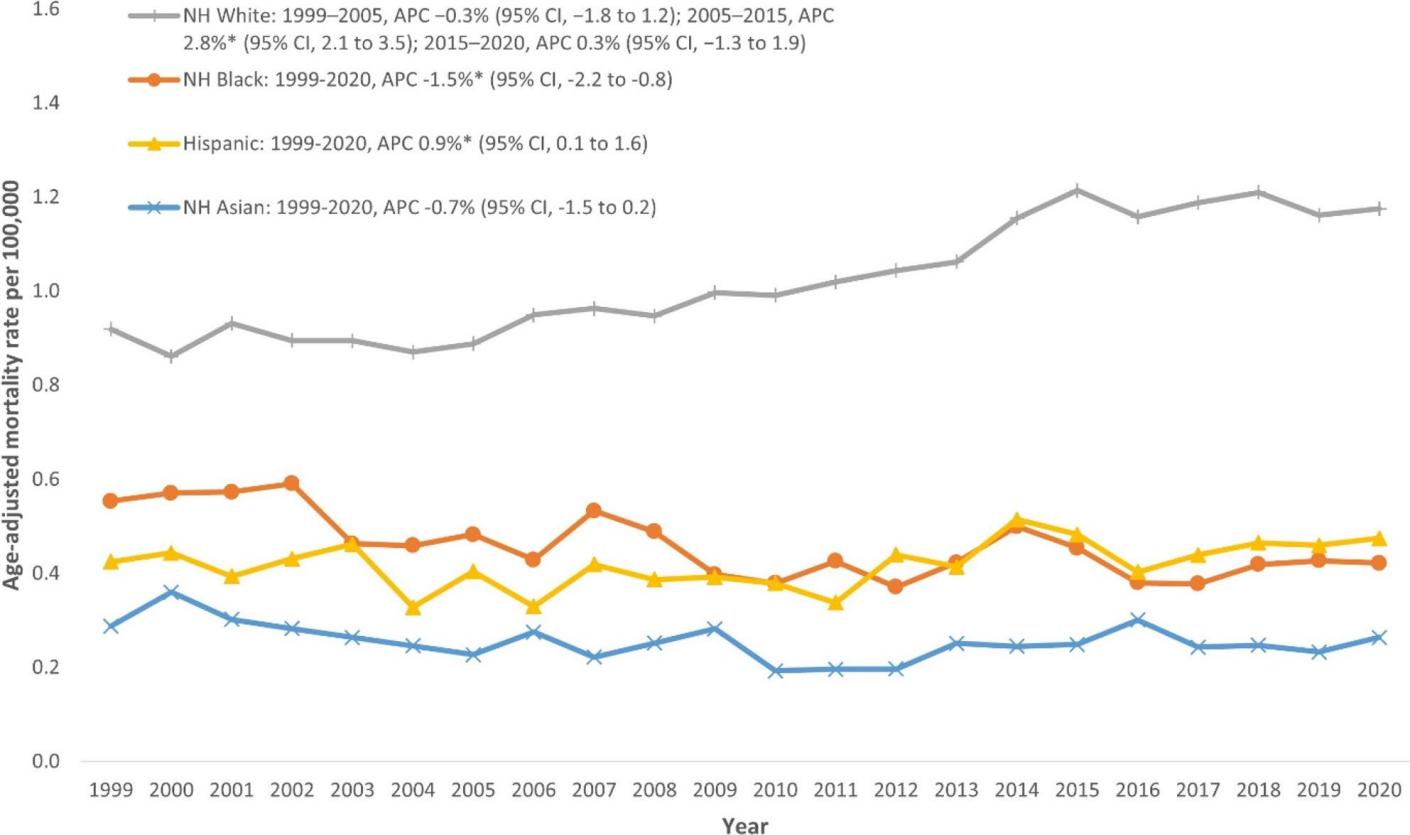
Trends in Nonmelanoma skin cancer-related age-adjusted mortality rate stratified by race in the United States, 1999–2020. *Indicates that the annual percentage change (APC) is significantly different from zero at α = 0.05. NH = non-Hispanic

AAMRs were highest among NH White individuals, followed by NH Black, Hispanic, and NH Asian individuals (overall AAMR NH White: 1.03; 95% CI: 1.02–1.04; NH Black: 0.44; 95% CI: 0.43–0.46; Hispanic: 0.42; 95% CI: 0.41–0.44; NH Asian: 0.23; 95% CI: 0.22–0.25). NH White individuals showed an initial declining trend from 1999 to 2005 (APC: -0.3%; 95% CI: -1.8 to 1.2), followed by a marked increase from 2005 to 2015 (APC: 2.8%; 95% CI: 2.1 to 3.5), then a slight stabilization between 2015 and 2020 (APC: 0.3%; 95% CI: -1.3 to 1.9). NH Black individuals showed a consistent declining trend throughout the entire study period (APC: -1.5%; 95% CI: -2.2 to -0.8). Hispanic individuals demonstrated a significant increasing trend (APC: 0.9%; 95% CI: 0.1 to 1.6), while NH Asian individuals exhibited a stable to slightly decreasing trend (APC: -0.7%; 95% CI: -1.5 to 0.2) (Fig. 2, Additional file 1: Supplemental Table 4). Because NH Asian AAMRs for 1999, 2002–2004, and 2008 required interpolation/extrapolation due to unreliable values, NH Asian trend estimates should be interpreted cautiously.

### NMSC-related AAMR stratified by age group

**Fig. 3:**
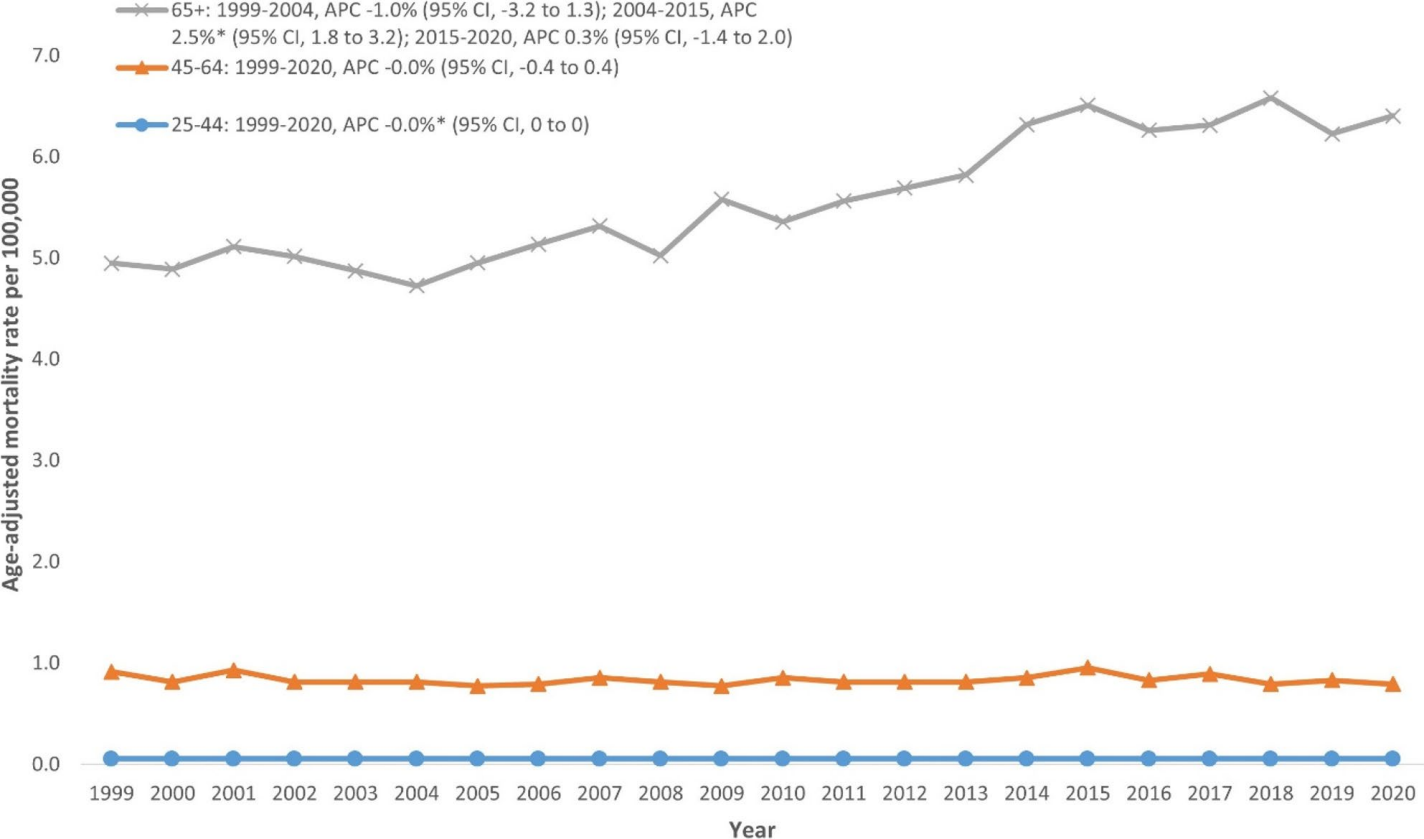
Trends in Nonmelanoma skin cancer-related age-adjusted mortality rate stratified by age in the United States, 1999–2020. *Indicates that the annual percentage change (APC) is significantly different from zero at α = 0.05

Individuals aged ≥ 65 years showed markedly higher AAMRs compared with younger age groups, with an overall AAMR of 5.63 per 100,000. This age group initially exhibited a decreasing trend from 1999 to 2004 (APC: -1.0%; 95% CI: -3.2 to 1.3), followed by a significant increase from 2004 to 2015 (APC: 2.5%; 95% CI: 1.8 to 3.2), and stabilization thereafter (2015–2020 APC: 0.3%; 95% CI: -1.4 to 2.0). Age groups 45–64 years and 25–44 years maintained relatively low and stable AAMRs across the entire period (Fig. 3, Additional file 1: Supplemental Table 5).

### NMSC-related AAMR stratified by geographic region

**Fig. 4:**
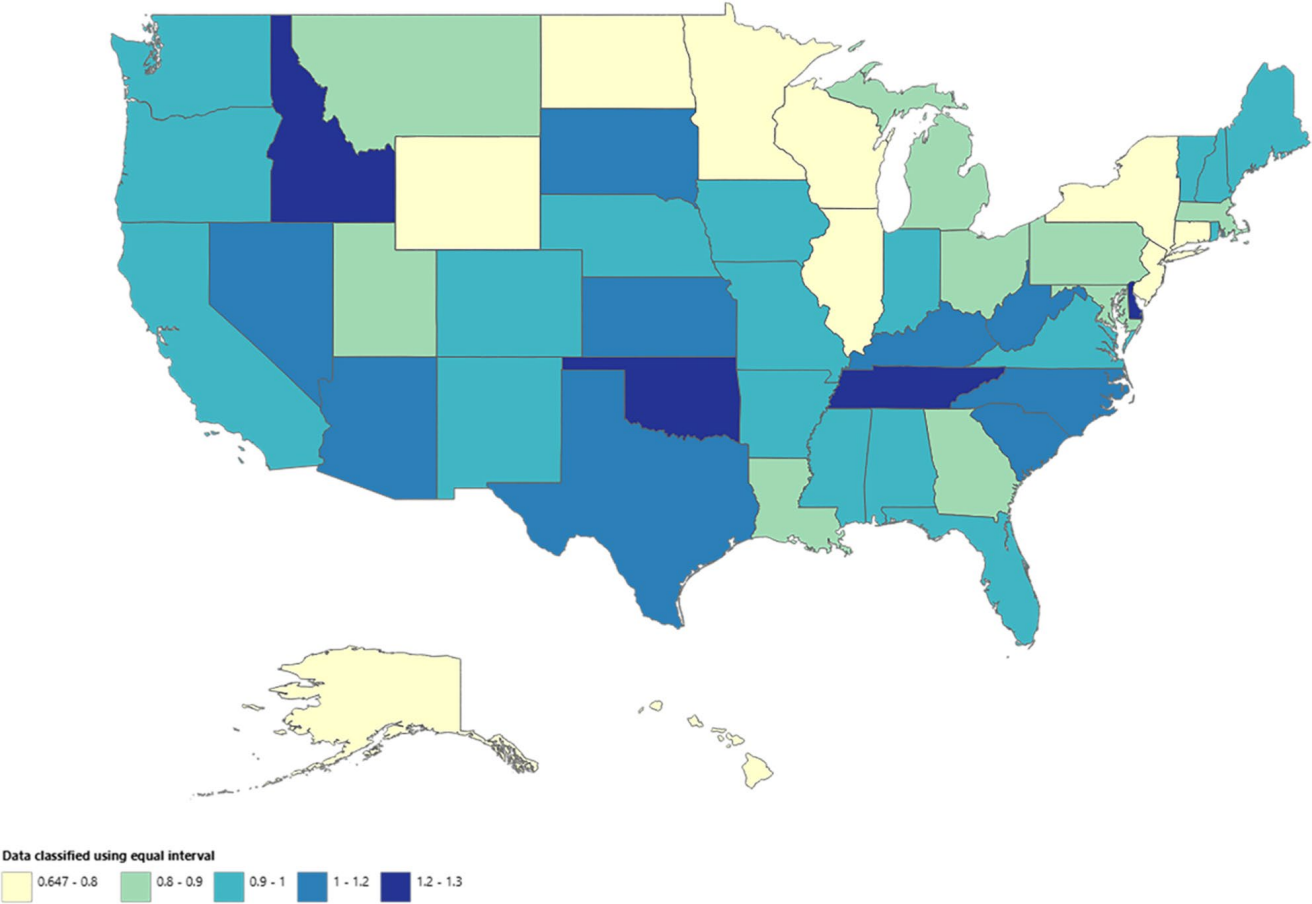
Nonmelanoma skin cancer-related age-adjusted mortality rate stratified by state in the United States, 1999–2020

**Fig. 5:**
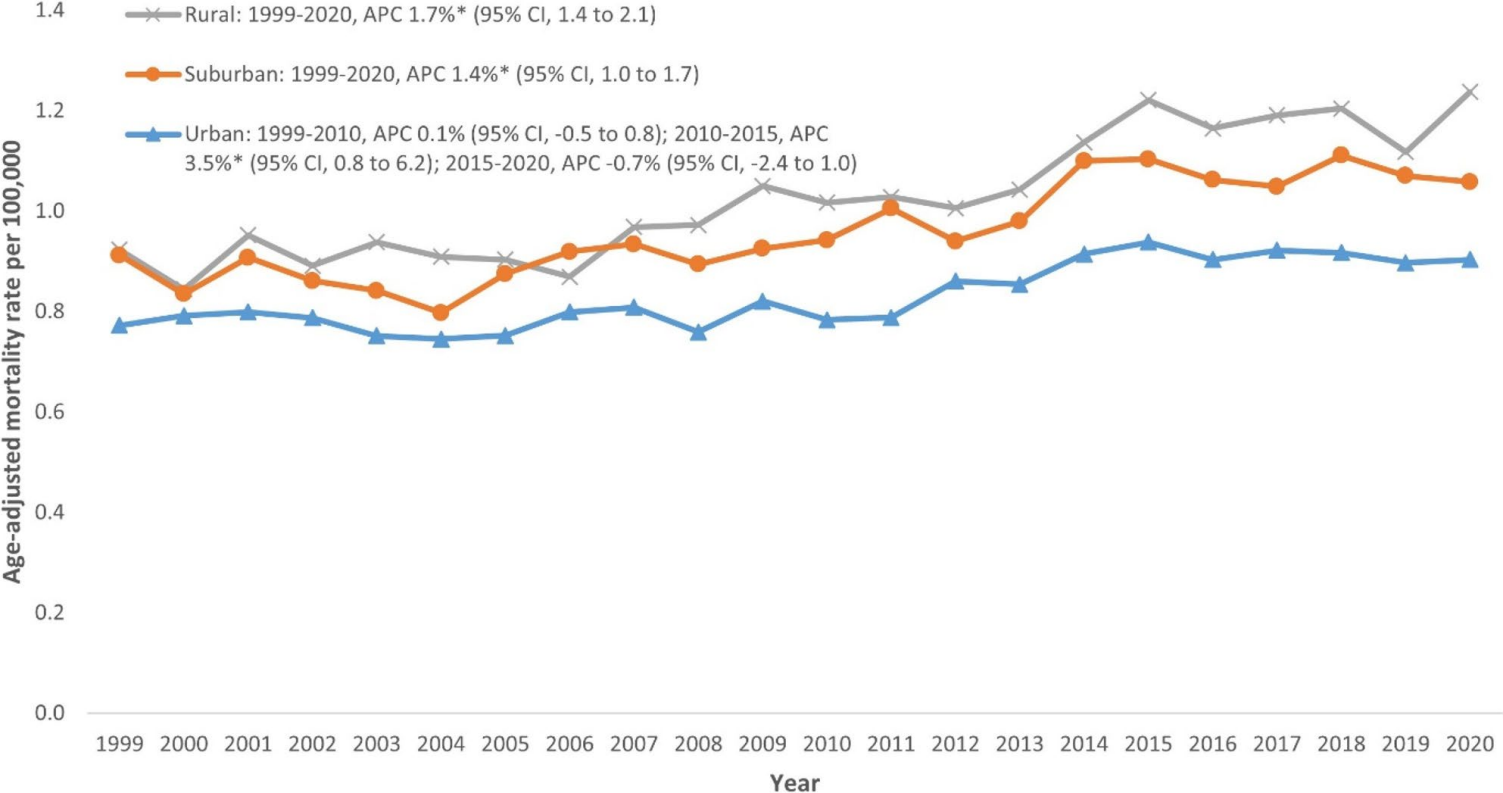
Trends in Nonmelanoma skin cancer-related age-adjusted mortality rate stratified by Urban-Rural Classification in the United States, 1999–2020. *Indicates that the annual percentage change (APC) is significantly different from zero at α = 0.05

State-level AAMRs ranged from 0.65 (95% CI: 0.52–0.80) in the District of Columbia to 1.29 (95% CI: 1.14–1.44) in Delaware. The top 10th percentile (Delaware, Tennessee, Oklahoma, Idaho, Arizona, Kentucky) had roughly double the AAMR of the bottom 10th percentile (District of Columbia, North Dakota, Alaska, New York, Hawaii, Connecticut) (Fig. 4; Additional file 1: Supplemental Table 6). On average over the study period, mortality was highest in the South (AAMR: 1.01; 95% CI: 1.00–1.02), followed by the West (AAMR: 0.96; 95% CI: 0.95–0.98), Midwest (AAMR: 0.86; 95% CI: 0.85–0.87), and Northeast (AAMR: 0.77; 95% CI: 0.76–0.79) (Additional file 1: Supplemental Table 7). Rural areas had consistently higher AAMRs (overall AAMR: 1.04; 95% CI: 1.02–1.06), followed by suburban (overall AAMR: 0.97; 95% CI: 0.95–0.98) and urban areas (overall AAMR: 0.83; 95% CI: 0.82–0.84). A significant rising trend was observed in rural (APC: 1.7%; 95% CI: 1.4 to 2.1) and suburban areas (APC: 1.4%; 95% CI: 1.0 to 1.7) throughout the study period. Urban areas displayed initial stability (1999–2010 APC: 0.1%; 95% CI: -0.5 to 0.8), followed by a significant rise from 2010 to 2015 (APC: 3.5%; 95% CI: 0.8 to 6.2) and subsequent stability from 2015 to 2020 (APC: -0.7%; 95% CI: -2.4 to 1.0) (Fig. 5, Additional file 1: Supplemental Table 8).

## Discussion

This nationwide analysis reveals that, despite advances in detection and treatment, NMSC mortality in the U.S. increased overall over the last two decades. After an initial dip in the early 2000s, age‑adjusted mortality rose significantly from 2004 onward, reaching 1.00 per 100,000 in 2020. We identified pronounced demographic disparities: men consistently had nearly threefold higher mortality than women, although female rates have escalated in recent years. NH White individuals experienced the highest mortality and the steepest increase over time, whereas NH Black individuals saw a modest decline in NMSC death rates. Mortality was overwhelmingly concentrated among older adults: by 2020, those aged ≥ 65 years had NMSC mortality more than eightfold higher than middle‑aged adults. Geographic patterns were also evident, with the Southern United States carrying the highest mortality burden and rural counties suffering greater NMSC mortality than suburban or urban areas.

Rising overall NMSC mortality, notably pronounced in those ≥ 65 years, likely reflects long‑latency effects of cumulative ultraviolet radiation over the life course and shifts in sun‑seeking behaviors (e.g., indoor tanning, intermittent intense sun exposure) that were prevalent in mid‑life decades ago [15–17]. Adoption of dermoscopy in the early 2000s substantially improved the diagnostic accuracy of keratinocyte carcinomas [18], potentially facilitating earlier tumor detection. However, high-risk cohorts exposed to intense UV radiation in prior decades have since aged into the most vulnerable group [16]. Indoor tanning and recreational sunbathing surged in popularity during the 1990s and early 2000s [19]. In 2009, the World Health Organization classified UV tanning devices as carcinogenic [20], and indoor tanning rates subsequently declined [21]. Yet irreversible damage from past UV exposure, responsible for roughly 90% of NMSC cases [22], likely contributed to the continued rise in mortality through the early 2010s. Our findings mirror trends in other high-UV settings: for example, in Australia, NMSC deaths have continued to increase despite decades of sun-safety campaigns [23]. Notably, although overall NMSC mortality continued to rise modestly through 2020, the rate of increase appeared to slow after 2015. This temporal change is likely multifactorial and may be coincident with the introduction of new systemic options for advanced disease (e.g., vismodegib in 2012 and PD-1 inhibitors for metastatic SCC by 2018) [24]. These advances, focused on inoperable or aggressive NMSCs (the primary cause of NMSC-related death), are a plausible correlative factor for the observed post-2015 deceleration in mortality growth. However, population-level mortality effects may lag behind approvals and should not be interpreted as evidence of causality.

Sex disparities in NMSC outcomes were striking and consistent. We found male mortality rates nearly three times higher than female rates, in line with prior national estimates and global patterns [25]. Men bear a disproportionate burden: epidemiologic analyses confirm that men have higher NMSC incidence, prevalence, and mortality than women [10]. Biological factors may contribute, as men appear more susceptible to UV‑induced immune suppression [26, 27], which could heighten their skin cancer risk. Consistent with these factors, nearly one-third of NMSC deaths globally have been attributed to occupational UV exposure [28]. This disparity underscores the need for targeted prevention in high-risk male groups (e.g., outdoor workers) to reduce excess male mortality.

Racial and ethnic patterns in NMSC mortality were also evident. NH White individuals accounted for the vast majority of NMSC deaths, with age-adjusted mortality more than double that of any other racial group. This aligns with the known epidemiology of skin cancer: NMSC is most common among fair‑skinned populations, and White individuals have far higher NMSC incidence than Black, Hispanic, or Asian individuals [1, 5]. Black Americans experienced the lowest NMSC mortality and even saw a modest decline over the study period, reflecting their much lower underlying NMSC incidence [5]. By contrast, in melanoma, White patients have the highest incidence, while minority patients often present with more advanced disease and poorer survival [29]. The comparatively low NMSC mortality in minority groups may reflect protective melanin pigmentation and historically limited UV exposure [30, 31]. However, important disparities persist: in patients of color, NMSC may present in less sun-exposed (including acral) sites and is often detected later, reflecting lower awareness and access-to-care barriers [30, 31]. Multiple factors, such as insurance status, access to care, cultural beliefs, and educational gaps, contribute to these disparities [30]. Our results underscore that NMSC mortality is not immune to health inequities; efforts to improve skin cancer awareness and access in communities of color remain important, even if absolute mortality rates in these groups are low.

Geographic disparities likely reflect environmental and healthcare system influences on NMSC outcomes. We observed the highest mortality rates in Southern states and the lowest in the Northeast, a north–south gradient that may reflect differences in ambient UV exposure and related behavioral patterns [5, 32]. State-level variation is likely multifactorial and may also reflect differences in rurality and access to dermatologic care, as well as underlying demographic composition; however, CDC WONDER does not capture state-level socioeconomic measures (e.g., poverty or unemployment) for adjustment in this analysis. We also found a clear rural–urban divide: rural counties had significantly higher NMSC mortality than suburban or urban areas. This rural excess may reflect an access gap, as rural residents may face difficulties obtaining timely evaluation and biopsy or early lesion removal, and higher poverty rates and lower insurance coverage could further delay diagnosis and treatment. Occupational sun exposure in rural settings (e.g., farming or construction) may also contribute [28, 33]. This rural excess emphasizes the need to bridge the care gap outside metropolitan centers through measures such as teledermatology outreach or mobile screening programs [34, 35].

Clinical and public health implications: Our findings reinforce the importance of strengthening both prevention and early detection of NMSC, especially in high-risk groups [9]. From a prevention standpoint, reducing UV exposure remains paramount. Preventive efforts should continue to focus on reducing UV exposure through policy measures (e.g., indoor tanning restrictions, particularly for minors) and environmental interventions such as sun‑safe school and workplace policies [36, 37]. Mass-media campaigns have shown a high return on investment, for example, an Australian program yielded $3.85 for every $1 spent [38]. Similar efforts in the U.S. could help instill lifelong sun protection habits [39]. Early detection strategies must also adapt to evolving epidemiology: for older adults, especially sun‑damaged men, regular total‑body skin examinations can facilitate prompt treatment of aggressive lesions [40]. Expanding opportunistic skin checks in primary care and community settings could improve early detection in regions lacking specialists [41]. Teledermatology and smartphone applications are promising adjuncts for triaging lesions in remote areas; some mobile apps show approximately 90% sensitivity for detecting malignancies [34]. Moreover, integrating artificial intelligence tools into screening may further enhance diagnostic accuracy and referral decisions [8]. Together, these digital tools may serve as a practical digital bridge to specialty input in rural settings, which had higher NMSC mortality in our analysis. By leveraging such technologies and targeted outreach (e.g., mobile skin clinics in high-mortality rural areas or subsidized screenings for high-risk seniors), resources can be allocated more equitably. A combined approach of public education, UV policy measures, and expanded screening could help reduce NMSC mortality and narrow the demographic gaps identified in this study.

Several limitations should be considered. First, our reliance on death certificate coding for cause of death may introduce misclassification bias; some fatalities coded as NMSC could represent metastatic disease from other primaries, while true NMSC deaths may go unrecorded. Second, CDC WONDER lacks tumor-level detail (including histologic subtype and stage), preventing us from distinguishing SCC from BCC mortality patterns and limiting interpretation of population-level mortality drivers and potential treatment-era effects. Third, our ecological, population‑level design precludes individual‑level inference and, combined with the suppression of low death counts for privacy, limits certain subgroup analyses (for example, state‑level trends among minority groups) and may obscure local hotspots. Fourth, to estimate suppressed age‑adjusted rates for NH Asian individuals, we used linear interpolation and extrapolation, an approach that may introduce estimation error, and therefore, inferences specific to NH Asian temporal patterns should be interpreted cautiously. Finally, key individual‑level risk factors (socioeconomic status, occupational UV exposure, and insurance coverage) are not captured in CDC WONDER, preventing adjustment for potential confounders in the observed mortality patterns. Accordingly, we could not quantify intersectional risk across combined subgroups (e.g., older NH White individuals residing in the South).

Future research should focus on: (1) establishing a dedicated national NMSC registry to improve epidemiologic tracking and guide targeted interventions; (2) evaluating the effectiveness of artificial intelligence-driven screening tools, particularly in rural and underserved populations; and (3) developing and implementing tailored sun-safety education and policy measures aimed specifically at high-risk populations, such as older adults, outdoor workers, and residents of regions with high UV exposure.

## Conclusion

Our analysis revealed a significant increase in U.S. NMSC mortality from 2004 to 2020, with marked disparities evident among older adults, men, NH White populations, rural counties, and Southern states. These findings reflect the cumulative impact of historical ultraviolet exposure and highlight gaps in skin cancer surveillance and healthcare accessibility. Our results underscore the need to prioritize targeted prevention strategies, regular screening, and resource allocation toward these high-risk groups to mitigate NMSC mortality.

## Supplementary Information

Supplementary Material 1.

## Data Availability

The datasets used and/or analyzed during the current study are available from the corresponding author on reasonable request. The data that support the findings of this study are derived from public domains, including the CDC WONDER database. Specific details of data extraction are disclosed in the manuscript, ensuring transparency and reproducibility.

## Abbreviations

NMSC: Nonmelanoma Skin Cancer
BCC: Basal Cell Carcinoma
SCC: Squamous Cell Carcinoma
CDC WONDER: Centers for Disease Control and Prevention’s Wide‑ranging Online Data for Epidemiologic Research
CDC: Centers for Disease Control and Prevention
ICD‑10: International Classification of Diseases, Tenth Revision
STROBE: Strengthening the Reporting of Observational Studies in Epidemiology
NH: Non‑Hispanic
NCHS: National Center for Health Statistics
U.S.: United States
AAMR: Age‑Adjusted Mortality Rate(s)
APC: Annual Percent Change
CI: Confidence Interval(s)
PD‑1: Programmed Cell Death Protein 1
p‑value: Probability Value
