# Supplementary material for: Demographic and regional trends in Nonmelanoma skin cancer mortality in the United States, 1999–2020
Source: BMC Cancer. 2026 Mar 31;26:605. doi: 10.1186/s12885-026-15953-z (PMC13169563; doi:10.1186/s12885-026-15953-z)
Supplement: Supplementary file 1 — Supplementary Material 1. [file 12885_2026_15953_MOESM1_ESM.docx]

**Article title:** Demographic and Regional Trends in Nonmelanoma Skin Cancer Mortality in the United States, 1999-2020

**Authors:**

Ahsan Raza Raja, MBBS^1^, Zoha Zahid Fazal, MBBS^2^, Aisha Sethi, MD, FAAD^3,4^

^1^Department of Medicine, Aga Khan University, Karachi, Sindh, Pakistan

^2^Stanford University School of Medicine, Palo Alto, CA, USA

^3^Department of Dermatology, Yale University School of Medicine, New Haven, CT, USA

^4^Yale Dermatology Global Health Program, New Haven, CT, USA

**Corresponding Author:**

Ahsan Raza Raja, MBBS

Department of Medicine, Aga Khan University

Stadium Road, P. O. Box 3500

Karachi, Sindh, Pakistan 74800

Email: [ahsan.raja@scholar.aku.edu](mailto:ahsan.raja@scholar.aku.edu)

**Supplemental Table 1**. Absolute Number of Nonmelanoma Skin Cancer-related Mortality, Stratified by Sex and Race in the United States, 1999-2020.

|  | **Deaths** | | | | | | |
| --- | --- | --- | --- | --- | --- | --- | --- |
| **Year** | **Overall** | **Female** | **Male** | **Non-Hispanic White** | **Non-Hispanic Black** | **Non-Hispanic Asian** | **Hispanic** |
| 1999 | 2315 | 725 | 1590 | 2080 | 145 | 17 | 60 |
| 2000 | 2252 | 698 | 1554 | 1997 | 150 | 25 | 70 |
| 2001 | 2450 | 792 | 1658 | 2196 | 152 | 24 | 63 |
| 2002 | 2391 | 745 | 1646 | 2141 | 159 | 10 | 70 |
| 2003 | 2397 | 748 | 1649 | 2159 | 126 | 19 | 79 |
| 2004 | 2349 | 704 | 1645 | 2122 | 136 | 18 | 62 |
| 2005 | 2453 | 721 | 1732 | 2195 | 142 | 23 | 77 |
| 2006 | 2627 | 741 | 1886 | 2378 | 131 | 28 | 71 |
| 2007 | 2773 | 853 | 1920 | 2478 | 170 | 24 | 90 |
| 2008 | 2714 | 789 | 1925 | 2437 | 163 | 16 | 85 |
| 2009 | 2931 | 860 | 2071 | 2646 | 136 | 34 | 97 |
| 2010 | 2935 | 816 | 2119 | 2660 | 137 | 24 | 92 |
| 2011 | 3084 | 844 | 2240 | 2794 | 151 | 28 | 93 |
| 2012 | 3212 | 896 | 2316 | 2898 | 134 | 31 | 126 |
| 2013 | 3354 | 870 | 2484 | 3011 | 163 | 35 | 121 |
| 2014 | 3734 | 1007 | 2727 | 3326 | 182 | 40 | 156 |
| 2015 | 3933 | 1095 | 2838 | 3538 | 170 | 43 | 160 |
| 2016 | 3855 | 1026 | 2829 | 3478 | 151 | 52 | 147 |
| 2017 | 3998 | 1139 | 2859 | 3600 | 162 | 44 | 164 |
| 2018 | 4167 | 1193 | 2974 | 3724 | 179 | 51 | 177 |
| 2019 | 4073 | 1206 | 2867 | 3624 | 185 | 51 | 189 |
| 2020 | 4226 | 1252 | 2974 | 3743 | 193 | 60 | 195 |
| **Total** | 68223 | 19720 | 48503 | 61225 | 3417 | 697 | 2444 |

**Supplemental Table 2.** Nonmelanoma Skin Cancer-related Age-Adjusted Mortality Rates per 10,000, Stratified by Place of Death in the United States, 1999-2020.

|  | **Deaths** | | | | |
| --- | --- | --- | --- | --- | --- |
| **Year** | **Medical facility** | **Home** | **Hospice facility** | **Nursing home/long-term care** | **Other** |
| 1999 | 695 | 878 | Missing | 593 | 139 |
| 2000 | 692 | 883 | Missing | 561 | 116 |
| 2001 | 759 | 931 | Missing | 609 | 151 |
| 2002 | 729 | 927 | Missing | 558 | 169 |
| 2003 | 721 | 891 | 11 | 591 | 172 |
| 2004 | 652 | 905 | 21 | 573 | 186 |
| 2005 | 687 | 940 | 88 | 572 | 155 |
| 2006 | 670 | 1023 | 121 | 667 | 145 |
| 2007 | 740 | 1045 | 162 | 659 | 154 |
| 2008 | 700 | 997 | 215 | 611 | 190 |
| 2009 | 653 | 1122 | 237 | 652 | 261 |
| 2010 | 635 | 1176 | 273 | 675 | 173 |
| 2011 | 703 | 1152 | 353 | 685 | 188 |
| 2012 | 716 | 1234 | 411 | 643 | 203 |
| 2013 | 645 | 1377 | 406 | 666 | 256 |
| 2014 | 724 | 1544 | 516 | 737 | 207 |
| 2015 | 755 | 1654 | 615 | 744 | 164 |
| 2016 | 720 | 1630 | 594 | 738 | 172 |
| 2017 | 773 | 1648 | 642 | 750 | 185 |
| 2018 | 774 | 1785 | 617 | 786 | 202 |
| 2019 | 722 | 1720 | 623 | 812 | 194 |
| 2020 | 656 | 2137 | 520 | 703 | 207 |
| **Total** | **15521** | **27599** | **6425** | **14585** | **3989** |

**Supplemental Table 3.** Overall and Sex‐Stratified Nonmelanoma Skin Cancer-related Age-Adjusted Mortality Rates per 10,000 in the United States, 1999-2020.

|  | **Age-Adjusted Rate (95% CI)** | | |
| --- | --- | --- | --- |
| **Year** | **Overall** | **Female** | **Male** |
| 1999 | 0.845 (0.81-0.879) | 0.424 (0.393-0.456) | 1.44 (1.367-1.512) |
| 2000 | 0.815 (0.781-0.849) | 0.405 (0.374-0.435) | 1.408 (1.336-1.479) |
| 2001 | 0.869 (0.835-0.904) | 0.473 (0.44-0.507) | 1.436 (1.365-1.507) |
| 2002 | 0.831 (0.797-0.864) | 0.437 (0.405-0.469) | 1.413 (1.343-1.483) |
| 2003 | 0.813 (0.78-0.846) | 0.415 (0.385-0.445) | 1.384 (1.316-1.453) |
| 2004 | 0.794 (0.762-0.826) | 0.39 (0.361-0.419) | 1.367 (1.299-1.435) |
| 2005 | 0.814 (0.782-0.847) | 0.406 (0.375-0.436) | 1.395 (1.328-1.462) |
| 2006 | 0.842 (0.809-0.874) | 0.398 (0.369-0.427) | 1.483 (1.415-1.552) |
| 2007 | 0.878 (0.845-0.911) | 0.454 (0.423-0.485) | 1.472 (1.405-1.539) |
| 2008 | 0.832 (0.801-0.864) | 0.412 (0.383-0.441) | 1.446 (1.38-1.512) |
| 2009 | 0.893 (0.861-0.926) | 0.461 (0.429-0.493) | 1.525 (1.458-1.592) |
| 2010 | 0.883 (0.851-0.915) | 0.408 (0.38-0.437) | 1.511 (1.446-1.577) |
| 2011 | 0.900 (0.868-0.933) | 0.405 (0.377-0.433) | 1.557 (1.491-1.623) |
| 2012 | 0.917 (0.885-0.949) | 0.440 (0.41-0.469) | 1.557 (1.492-1.621) |
| 2013 | 0.933 (0.901-0.965) | 0.403 (0.376-0.431) | 1.646 (1.58-1.712) |
| 2014 | 1.005 (0.972-1.037) | 0.466 (0.436-0.496) | 1.739 (1.672-1.805) |
| 2015 | 1.051 (1.017-1.084) | 0.496 (0.466-0.526) | 1.764 (1.698-1.831) |
| 2016 | 0.993 (0.961-1.024) | 0.459 (0.429-0.488) | 1.718 (1.653-1.782) |
| 2017 | 1.012 (0.98-1.044) | 0.486 (0.457-0.515) | 1.682 (1.619-1.745) |
| 2018 | 1.024 (0.993-1.056) | 0.523 (0.492-0.554) | 1.716 (1.653-1.778) |
| 2019 | 0.988 (0.957-1.019) | 0.497 (0.469-0.526) | 1.611 (1.55-1.671) |
| 2020 | 1.002 (0.971-1.032) | 0.520 (0.49-0.549) | 1.657 (1.596-1.718) |

**Supplemental Table 4.** Nonmelanoma Skin Cancer-related Age-Adjusted Mortality Rates per 10,000, Stratified by Race in the United States, 1999-2020.

|  | **Age-Adjusted Rate (95% CI)** | | | | |
| --- | --- | --- | --- | --- | --- |
| **Year** | **Non-Hispanic White** | **Non-Hispanic Black** | **Non-Hispanic Asian** | **Non-Hispanic American Indian** | **Hispanic** |
| 1999 | 0.919 (0.879-0.958) | 0.554 (0.461-0.646) | Unreliable^a^ (0.148-0.42) | Suppressed^a^ | 0.425 (0.319-0.555) |
| 2000 | 0.861 (0.823-0.899) | 0.571 (0.479-0.664) | 0.36 (0.223-0.551) | Suppressed^a^ | 0.444 (0.34-0.569) |
| 2001 | 0.931 (0.892-0.97) | 0.573 (0.48-0.666) | 0.302 (0.189-0.458) | Suppressed^a^ | 0.394 (0.296-0.512) |
| 2002 | 0.895 (0.857-0.933) | 0.591 (0.497-0.684) | Unreliable^a^ (0.047-0.197) | Suppressed^a^ | 0.431 (0.332-0.55) |
| 2003 | 0.894 (0.856-0.932) | 0.463 (0.38-0.545) | Unreliable^a^ (0.121-0.345) | Suppressed^a^ | 0.462 (0.361-0.583) |
| 2004 | 0.87 (0.833-0.907) | 0.459 (0.38-0.539) | Unreliable^a^ (0.129-0.355) | Suppressed^a^ | 0.328 (0.245-0.43) |
| 2005 | 0.888 (0.851-0.926) | 0.483 (0.401-0.565) | 0.227 (0.138-0.35) | Unreliable^a^ (0.285-1.298) | 0.404 (0.313-0.513) |
| 2006 | 0.949 (0.91-0.987) | 0.429 (0.353-0.504) | 0.275 (0.18-0.403) | Unreliable^a^ (0.416-1.594) | 0.33 (0.254-0.423) |
| 2007 | 0.963 (0.925-1.001) | 0.533 (0.449-0.616) | 0.222 (0.139-0.336) | Suppressed^a^ | 0.419 (0.332-0.523) |
| 2008 | 0.947 (0.909-0.985) | 0.489 (0.411-0.567) | Unreliable^a^ (0.074-0.218) | Suppressed^a^ | 0.387 (0.304-0.485) |
| 2009 | 0.997 (0.959-1.035) | 0.398 (0.328-0.468) | 0.282 (0.192-0.401) | Unreliable^a^ (0.426-1.44) | 0.392 (0.313-0.484) |
| 2010 | 0.991 (0.953-1.029) | 0.379 (0.312-0.445) | 0.193 (0.121-0.292) | Unreliable^a^ (0.521-1.598) | 0.379 (0.301-0.471) |
| 2011 | 1.019 (0.981-1.057) | 0.426 (0.355-0.497) | 0.196 (0.127-0.289) | Unreliable^a^ (0.314-1.062) | 0.338 (0.269-0.419) |
| 2012 | 1.044 (1.006-1.083) | 0.371 (0.306-0.437) | 0.197 (0.131-0.285) | Unreliable^a^ (0.29-1.113) | 0.44 (0.358-0.522) |
| 2013 | 1.062 (1.023-1.1) | 0.423 (0.355-0.491) | 0.251 (0.174-0.351) | Unreliable^a^ (0.292-1.045) | 0.414 (0.337-0.492) |
| 2014 | 1.155 (1.115-1.195) | 0.5 (0.424-0.575) | 0.245 (0.173-0.336) | Unreliable^a^ (0.397-1.274) | 0.515 (0.432-0.598) |
| 2015 | 1.214 (1.173-1.254) | 0.454 (0.383-0.525) | 0.249 (0.179-0.338) | Unreliable^a^ (0.313-1.006) | 0.483 (0.404-0.561) |
| 2016 | 1.158 (1.119-1.198) | 0.38 (0.317-0.444) | 0.301 (0.223-0.398) | Unreliable^a^ (0.309-1.046) | 0.403 (0.333-0.474) |
| 2017 | 1.188 (1.149-1.228) | 0.378 (0.317-0.439) | 0.243 (0.175-0.33) | Unreliable^a^ (0.164-0.681) | 0.439 (0.369-0.509) |
| 2018 | 1.21 (1.17-1.249) | 0.419 (0.355-0.482) | 0.247 (0.182-0.328) | Unreliable^a^ (0.424-1.166) | 0.465 (0.394-0.535) |
| 2019 | 1.161 (1.123-1.2) | 0.427 (0.364-0.491) | 0.233 (0.173-0.307) | Unreliable^a^ (0.293-0.899) | 0.46 (0.392-0.529) |
| 2020 | 1.175 (1.136-1.213) | 0.422 (0.36-0.484) | 0.264 (0.2-0.341) | 0.744 (0.461-1.138) | 0.475 (0.406-0.544) |

^a^ Mortality data suppressed/unreliable due to CDC privacy guidelines related to low death counts.

**Supplemental Table 5.** Nonmelanoma Skin Cancer-related Age-Adjusted Mortality Rates per 10,000, Stratified by age groups in the United States, 1999-2020.

|  | **Age-Adjusted Rate (95% CI)** | | | |
| --- | --- | --- | --- | --- |
| **Year** | **Age <25 years** | **Age 25-44 years** | **Age 45-64 years** | **Age 65+ years** |
| 1999 | Suppressed^a^ | 0.055 (0.042-0.07) | 0.914 (0.837-0.992) | 4.95 (4.715-5.184) |
| 2000 | Suppressed^a^ | 0.055 (0.04-0.073) | 0.814 (0.743-0.885) | 4.889 (4.656-5.122) |
| 2001 | Suppressed^a^ | 0.055 (0.04-0.073) | 0.932 (0.857-1.007) | 5.112 (4.876-5.349) |
| 2002 | Suppressed^a^ | 0.055 (0.041-0.071) | 0.814 (0.746-0.882) | 5.015 (4.781-5.248) |
| 2003 | Suppressed^a^ | 0.055 (0.041-0.071) | 0.814 (0.747-0.882) | 4.874 (4.646-5.101) |
| 2004 | Suppressed^a^ | 0.055 (0.039-0.074) | 0.814 (0.748-0.881) | 4.725 (4.501-4.948) |
| 2005 | Suppressed^a^ | 0.055 (0.04-0.072) | 0.775 (0.711-0.839) | 4.952 (4.725-5.178) |
| 2006 | Suppressed^a^ | 0.055 (0.039-0.075) | 0.793 (0.732-0.854) | 5.138 (4.909-5.367) |
| 2007 | Suppressed^a^ | 0.055 (0.039-0.074) | 0.854 (0.789-0.918) | 5.317 (5.087-5.547) |
| 2008 | Suppressed^a^ | 0.055 (0.039-0.074) | 0.814 (0.753-0.876) | 5.025 (4.804-5.246) |
| 2009 | Suppressed^a^ | 0.055 (0.039-0.075) | 0.775 (0.715-0.835) | 5.579 (5.347-5.811) |
| 2010 | Suppressed^a^ | 0.055 (0.037-0.078) | 0.854 (0.79-0.917) | 5.357 (5.132-5.582) |
| 2011 | Suppressed^a^ | 0.055 (0.037-0.078) | 0.814 (0.753-0.875) | 5.565 (5.339-5.791) |
| 2012 | Suppressed^a^ | 0.055 (0.038-0.075) | 0.814 (0.753-0.875) | 5.694 (5.468-5.921) |
| 2013 | Suppressed^a^ | 0.055 (0.039-0.074) | 0.814 (0.754-0.875) | 5.82 (5.594-6.047) |
| 2014 | Suppressed^a^ | 0.055 (0.039-0.074) | 0.854 (0.793-0.914) | 6.32 (6.088-6.552) |
| 2015 | Suppressed^a^ | 0.055 (0.038-0.076) | 0.954 (0.888-1.019) | 6.51 (6.277-6.743) |
| 2016 | Suppressed^a^ | 0.055 (0.038-0.075) | 0.832 (0.774-0.891) | 6.262 (6.037-6.488) |
| 2017 | Suppressed^a^ | 0.055 (0.037-0.078) | 0.893 (0.83-0.956) | 6.313 (6.09-6.536) |
| 2018 | Suppressed^a^ | 0.055 (0.039-0.074) | 0.793 (0.736-0.85) | 6.582 (6.357-6.807) |
| 2019 | Suppressed^a^ | 0.055 (0.036-0.079) | 0.832 (0.773-0.892) | 6.228 (6.012-6.443) |
| 2020 | Suppressed^a^ | 0.055 (0.041-0.071) | 0.793 (0.735-0.851) | 6.405 (6.188-6.622) |

^a^ Mortality data suppressed due to CDC privacy guidelines related to low death counts.

**Supplemental Table 6.** Nonmelanoma Skin Cancer-related Age-Adjusted Mortality Rates per 10,000, Stratified by State in the United States, 1999-2020.

| **State** | **Rank** | **Percentile** | **Age-Adjusted Rate (95% CI)** |
| --- | --- | --- | --- |
| District of Columbia | 1 | 0 | 0.647 (0.516-0.801) |
| North Dakota | 2 | 2 | 0.651 (0.533-0.768) |
| Alaska | 3 | 4 | 0.653 (0.503-0.834) |
| New York | 4 | 6 | 0.68 (0.657-0.704) |
| Hawaii | 5 | 8 | 0.712 (0.624-0.8) |
| Connecticut | 6 | 10 | 0.725 (0.671-0.779) |
| New Jersey | 7 | 12 | 0.733 (0.697-0.769) |
| Minnesota | 8 | 14 | 0.738 (0.691-0.785) |
| Illinois | 9 | 16 | 0.744 (0.713-0.775) |
| Wyoming | 10 | 18 | 0.766 (0.614-0.919) |
| Wisconsin | 11 | 20 | 0.798 (0.751-0.844) |
| Michigan | 12 | 22 | 0.805 (0.77-0.841) |
| Utah | 13 | 24 | 0.808 (0.725-0.891) |
| Pennsylvania | 14 | 26 | 0.812 (0.782-0.841) |
| Louisiana | 15 | 28 | 0.824 (0.768-0.88) |
| Montana | 16 | 30 | 0.825 (0.715-0.935) |
| Maryland | 17 | 32 | 0.841 (0.791-0.891) |
| Ohio | 18 | 34 | 0.845 (0.812-0.878) |
| Georgia | 19 | 36 | 0.865 (0.823-0.908) |
| Massachusetts | 20 | 38 | 0.875 (0.83-0.919) |
| Alabama | 21 | 40 | 0.908 (0.853-0.963) |
| Virginia | 22 | 42 | 0.911 (0.866-0.956) |
| Oregon | 23 | 44 | 0.921 (0.861-0.982) |
| Mississippi | 24 | 46 | 0.929 (0.857-1.001) |
| Missouri | 25 | 48 | 0.931 (0.882-0.979) |
| California | 26 | 50 | 0.939 (0.917-0.96) |
| Iowa | 27 | 52 | 0.951 (0.885-1.016) |
| Rhode Island | 28 | 52 | 0.951 (0.839-1.063) |
| New Mexico | 29 | 56 | 0.953 (0.865-1.041) |
| Vermont | 30 | 58 | 0.956 (0.805-1.108) |
| Nebraska | 31 | 60 | 0.965 (0.876-1.055) |
| Arkansas | 32 | 62 | 0.969 (0.898-1.04) |
| Colorado | 33 | 64 | 0.979 (0.918-1.041) |
| Washington | 34 | 66 | 0.983 (0.933-1.033) |
| Maine | 35 | 68 | 0.986 (0.887-1.085) |
| New Hampshire | 36 | 70 | 0.988 (0.881-1.096) |
| Indiana | 37 | 72 | 0.993 (0.943-1.043) |
| Florida | 38 | 74 | 0.995 (0.969-1.021) |
| South Dakota | 39 | 76 | 1.01 (0.875-1.146) |
| North Carolina | 40 | 78 | 1.03 (0.987-1.072) |
| Kansas | 41 | 80 | 1.065 (0.989-1.141) |
| Nevada | 42 | 80 | 1.065 (0.979-1.152) |
| Texas | 43 | 84 | 1.069 (1.04-1.098) |
| South Carolina | 44 | 86 | 1.094 (1.032-1.156) |
| West Virginia | 45 | 88 | 1.103 (1.012-1.193) |
| Kentucky | 46 | 90 | 1.115 (1.05-1.18) |
| Arizona | 47 | 92 | 1.122 (1.069-1.176) |
| Idaho | 48 | 94 | 1.222 (1.105-1.338) |
| Oklahoma | 49 | 96 | 1.275 (1.201-1.349) |
| Tennessee | 50 | 98 | 1.281 (1.223-1.338) |
| Delaware | 51 | 100 | 1.292 (1.144-1.441) |

**Supplemental Table 7.** Nonmelanoma Skin Cancer-related Age-Adjusted Mortality Rates per 10,000, Stratified by Census Region in the United States, 1999-2020.

|  | **Age-Adjusted Rate (95% CI)** | | | |
| --- | --- | --- | --- | --- |
| **Year** | **Northeast** | **Midwest** | **South** | **West** |
| 1999 | 0.766 (0.694-0.839) | 0.785 (0.717-0.853) | 0.968 (0.906-1.03) | 0.800 (0.724-0.875) |
| 2000 | 0.654 (0.588-0.72) | 0.756 (0.689-0.823) | 0.899 (0.84-0.958) | 0.882 (0.804-0.961) |
| 2001 | 0.755 (0.685-0.825) | 0.783 (0.716-0.85) | 0.994 (0.932-1.056) | 0.818 (0.743-0.892) |
| 2002 | 0.701 (0.634-0.768) | 0.766 (0.7-0.832) | 0.962 (0.901-1.024) | 0.825 (0.752-0.899) |
| 2003 | 0.712 (0.644-0.78) | 0.767 (0.702-0.832) | 0.912 (0.853-0.971) | 0.841 (0.767-0.915) |
| 2004 | 0.703 (0.636-0.77) | 0.717 (0.653-0.78) | 0.884 (0.827-0.941) | 0.815 (0.743-0.887) |
| 2005 | 0.690 (0.624-0.755) | 0.767 (0.703-0.832) | 0.871 (0.815-0.927) | 0.845 (0.772-0.918) |
| 2006 | 0.746 (0.678-0.813) | 0.812 (0.745-0.879) | 0.919 (0.862-0.976) | 0.834 (0.764-0.905) |
| 2007 | 0.735 (0.668-0.802) | 0.817 (0.752-0.883) | 0.963 (0.906-1.021) | 0.892 (0.82-0.965) |
| 2008 | 0.666 (0.603-0.73) | 0.83 (0.765-0.896) | 0.913 (0.858-0.968) | 0.878 (0.807-0.949) |
| 2009 | 0.794 (0.725-0.863) | 0.776 (0.712-0.84) | 0.943 (0.888-0.999) | 1.018 (0.943-1.094) |
| 2010 | 0.757 (0.689-0.824) | 0.830 (0.765-0.894) | 0.918 (0.864-0.972) | 0.915 (0.844-0.986) |
| 2011 | 0.746 (0.681-0.811) | 0.869 (0.803-0.935) | 0.966 (0.911-1.021) | 0.945 (0.873-1.016) |
| 2012 | 0.811 (0.743-0.88) | 0.839 (0.774-0.904) | 0.971 (0.917-1.025) | 0.957 (0.887-1.027) |
| 2013 | 0.764 (0.697-0.831) | 0.84 (0.776-0.904) | 1.042 (0.986-1.097) | 0.984 (0.914-1.055) |
| 2014 | 0.835 (0.767-0.904) | 0.95 (0.882-1.018) | 1.127 (1.07-1.184) | 1.015 (0.945-1.086) |
| 2015 | 0.894 (0.823-0.964) | 0.972 (0.904-1.039) | 1.124 (1.068-1.18) | 1.070 (0.999-1.142) |
| 2016 | 0.866 (0.797-0.936) | 0.929 (0.863-0.995) | 1.083 (1.028-1.138) | 1.043 (0.973-1.113) |
| 2017 | 0.847 (0.779-0.914) | 0.904 (0.839-0.969) | 1.132 (1.077-1.188) | 1.057 (0.988-1.127) |
| 2018 | 0.805 (0.74-0.869) | 0.987 (0.92-1.054) | 1.112 (1.058-1.165) | 1.114 (1.044-1.184) |
| 2019 | 0.795 (0.731-0.858) | 0.839 (0.778-0.901) | 1.107 (1.054-1.16) | 1.037 (0.971-1.104) |
| 2020 | 0.788 (0.725-0.852) | 0.966 (0.9-1.031) | 1.086 (1.034-1.138) | 1.079 (1.013-1.146) |

**Supplemental Table 8.** Nonmelanoma Skin Cancer-related Age-Adjusted Mortality Rates per 10,000, Stratified by Urban-Rural Classification in the United States, 1999-2020.

|  | **Age-Adjusted Rate (95% CI)** | | |
| --- | --- | --- | --- |
| **Year** | **Urban** | **Suburban** | **Rural** |
| 1999 | 0.772 (0.726-0.818) | 0.912 (0.847-0.977) | 0.923 (0.839-1.007) |
| 2000 | 0.791 (0.744-0.838) | 0.835 (0.774-0.896) | 0.844 (0.765-0.924) |
| 2001 | 0.799 (0.752-0.845) | 0.907 (0.844-0.97) | 0.952 (0.869-1.036) |
| 2002 | 0.787 (0.741-0.832) | 0.861 (0.799-0.922) | 0.891 (0.811-0.972) |
| 2003 | 0.751 (0.708-0.795) | 0.841 (0.781-0.901) | 0.938 (0.855-1.02) |
| 2004 | 0.745 (0.701-0.789) | 0.797 (0.738-0.855) | 0.909 (0.829-0.988) |
| 2005 | 0.752 (0.708-0.795) | 0.875 (0.814-0.936) | 0.903 (0.822-0.984) |
| 2006 | 0.799 (0.755-0.844) | 0.919 (0.858-0.98) | 0.869 (0.791-0.946) |
| 2007 | 0.808 (0.764-0.852) | 0.934 (0.874-0.994) | 0.968 (0.886-1.05) |
| 2008 | 0.759 (0.716-0.801) | 0.894 (0.835-0.952) | 0.972 (0.891-1.052) |
| 2009 | 0.82 (0.776-0.864) | 0.925 (0.865-0.984) | 1.050 (0.966-1.134) |
| 2010 | 0.783 (0.74-0.825) | 0.942 (0.882-1.002) | 1.017 (0.935-1.099) |
| 2011 | 0.788 (0.746-0.831) | 1.005 (0.945-1.066) | 1.028 (0.946-1.11) |
| 2012 | 0.86 (0.817-0.903) | 0.94 (0.882-0.999) | 1.006 (0.926-1.086) |
| 2013 | 0.854 (0.811-0.897) | 0.980 (0.921-1.038) | 1.043 (0.961-1.126) |
| 2014 | 0.914 (0.87-0.957) | 1.100 (1.04-1.161) | 1.137 (1.05-1.223) |
| 2015 | 0.938 (0.894-0.981) | 1.103 (1.042-1.163) | 1.221 (1.134-1.309) |
| 2016 | 0.903 (0.86-0.946) | 1.062 (1.003-1.121) | 1.165 (1.08-1.25) |
| 2017 | 0.922 (0.88-0.964) | 1.049 (0.992-1.107) | 1.191 (1.107-1.275) |
| 2018 | 0.917 (0.876-0.958) | 1.111 (1.053-1.17) | 1.204 (1.119-1.288) |
| 2019 | 0.897 (0.856-0.937) | 1.07 (1.012-1.127) | 1.118 (1.036-1.201) |
| 2020 | 0.903 (0.863-0.944) | 1.058 (1.002-1.114) | 1.237 (1.154-1.321) |
